# Supplementary material for: The genome sequence of the White-barred Clearwing, Synanthedon spheciformis (Denis & Schiffermüller), 1775 (Lepidoptera: Sesiidae)
Source: Wellcome Open Res. 2025 Jul 28;10:373. [Version 1] doi: 10.12688/wellcomeopenres.24613.1 (PMC12449690; doi:10.12688/wellcomeopenres.24613.1)
Supplement: Peer review history

# Reviewer report: Reviewer response for version 1

**DOI:** 10.21956/wellcomeopenres.27123.r131954

**Published:** 2025-09-30

**Authors:** Teodora Staykova

**Affiliations:** 1 Plovdiv University, Plovdiv, Bulgaria

The genome of *Synanthedon speciformis* (Lepidoptera: Sesiidae) was studied based on a female individual. The methods used are described in detail. Two haplotypes were identified. The majority of haplotype 1 (99.88%) is scaffolded in 31 chromosomal pseudomolecules, of which 30 are autosomes and one sex chromosome. The sex Z chromosome was identified by coverage and alignment with the genome of Synanthedon formicaeformis (GCA_945859745.1). No second sex chromosome was identified during the study, leading to the conclusion that female heterogamety of the ZO type is observed in this species.

 In addition to presenting new interesting genetic data for the species, the study is also significant because it provides a basis for studying population genetic characteristics, host plant specialization and the evolutionary history of the Sesiidae family.

Are sufficient details of methods and materials provided to allow replication by others?

Yes

Is the rationale for creating the dataset(s) clearly described?

Yes

Are the datasets clearly presented in a useable and accessible format?

Yes

Are the protocols appropriate and is the work technically sound?

Yes

Reviewer Expertise:

Genetics, Population Genetics, Genetics of Bombyx mori

I confirm that I have read this submission and believe that I have an appropriate level of expertise to confirm that it is of an acceptable scientific standard.

# Reviewer report: Reviewer response for version 1

**DOI:** 10.21956/wellcomeopenres.27123.r131949

**Published:** 2025-09-19

**Authors:** Jason Charamis

**Affiliations:** 1 Foundation for Research and Technology - Hellas, Irákleion, Greece

This manuscript describes the genome assembly of the White-barred Clearwing, *Synanthedon spheciformis *. The work is technically sound, and the authors have provided a very good dataset and a good rationale about the usefulness of this resource in future studies. I have no further comments or suggestions to make; the manuscript is ready for indexing.

Are sufficient details of methods and materials provided to allow replication by others?

Yes

Is the rationale for creating the dataset(s) clearly described?

Yes

Are the datasets clearly presented in a useable and accessible format?

Yes

Are the protocols appropriate and is the work technically sound?

Yes

Reviewer Expertise:

Arthropod Comparative Genomics

I confirm that I have read this submission and believe that I have an appropriate level of expertise to confirm that it is of an acceptable scientific standard.

# Reviewer report: Reviewer response for version 1

**DOI:** 10.21956/wellcomeopenres.27123.r130965

**Published:** 2025-09-16

**Authors:** Shova Mishra

**Affiliations:** 1 University of Florida, Gainesville, USA; 2 North Carolina State University, Raleigh, USA

The article presents a genome sequence of *Synanthedon spheciformis,* including two haplotypes  with different genome sizes. The manuscript is well written with clear introduction, methods, and results. Associated data sets  are made publicly available which is very useful for the scientific community. 

 The paper describes the RNA-seq method briefly. However, there is no corresponding results presented,   nor the data is uploaded. Keeping this in mind, it may not be necessary to include RNA-seq section in the method section.

Are sufficient details of methods and materials provided to allow replication by others?

Yes

Is the rationale for creating the dataset(s) clearly described?

Yes

Are the datasets clearly presented in a useable and accessible format?

Yes

Are the protocols appropriate and is the work technically sound?

Yes

Reviewer Expertise:

Nematology, Genomics, Transcriptomics, Nematicide resitance

I confirm that I have read this submission and believe that I have an appropriate level of expertise to confirm that it is of an acceptable scientific standard.
